# A three-snoRNA signature: SNORD15A, SNORD35B and SNORD60 as novel biomarker for renal cell carcinoma

**DOI:** 10.1186/s12935-023-02978-8

**Published:** 2023-07-13

**Authors:** Yue Zhang, Xiaoling Shang, Miao Yu, Zhao Bi, Kangyu Wang, Qianru Zhang, Li Xie, Xianrang Song, Xingguo Song

**Affiliations:** 1 Department of Clinical Laboratory, Shandong Cancer Hospital and Institute, Shandong First Medical University, Shandong Academy of Medical Sciences, 440 Ji-Yan Road, Jinan, 250117 Shandong Province PR China; 2 Shanghai Pudong New Area Center for Disease Control and Prevention, 3039 Zhangyang Road, Shanghai, China; 3 Department of Clinical Laboratory, Cheeloo College of Medicine, Shandong Provincial Third Hospital, Shandong University, Jinan, Shandong PR China; 4 Shandong Provincial Key Laboratory of Radiation Oncology, Shandong Cancer Hospital and Institute, Shandong First Medical University, Shandong Academy of Medical Sciences, Jinan, Shandong PR China

**Keywords:** SnoRNAs, SNORD15A, SNORD35B, SNORD60, Renal cell carcinoma, Urinary sediment, Biomarkers

## Abstract

**Background:**

Accumulating evidence has confirmed the role of snoRNAs in a variety of cancer, but rare in renal cell carcinoma (RCC). This study aims to clarify the role of snoRNAs in RCC tumorigenesis and their potential as novel tumor biomarkers.

**Materials and methods:**

The snoRNA expression matrix was obtained from the public TCGA and SNORic databases. SNORD15A, SNORD35B and SNORD60 were selected and validated by qPCR, then analyzed combined with related clinical factors using T-test and ROC curve.

**Results:**

All three snoRNAs: SNORD15A, SNORD35B and SNORD60 were significantly upregulated in cancer tissues compared to adjacent tissues from TCGA or FFPE detection. These three snoRNAs were also increased in urinary sediment (US) of RCC as well as the early-stage RCC patients compared with the healthy controls. In addition, RNase stability experiments confirmed their stable existence in US. Meanwhile, the ROC curve shows that SNORD15A, SNORD35B and SNORD60 could effectively distinguish RCC (AUC = 0.7421) and early-stage RCC (AUC = 0.7465) from healthy individuals.

**Conclusion:**

SNORD15A, SNORD35B and SNORD60 were upregulated in tissues and US of RCC, serving as novel potential biomarkers for RCC diagnosis.

## Introduction

Renal cell carcinoma (RCC), the common malignant tumor of the urinary system, mainly includes clear cell RCC (ccRCC), papillary RCC (pRCC), and chromophobe RCC (chRCC) [1]. Recently, the incidence of RCC has gradually increased, with an incidence of 400,000 new cases worldwide every year. As the symptoms of RCC are not obvious at the early stage, most patients have reached a late stage when confirmed and were not sensitive to radiotherapy, chemotherapy, or targeted therapy, causing the 5-year survival rate of RCC patients, especially those with metastasis, was not hopeful [2]. The key to improving the prognosis and the survival rate of RCC patients was early diagnosis and treatment [3]. However, there is still a lack of satisfactory early diagnostic biomarkers for RCC despite the continuous progress of diagnostic technology.

Small nucleolar RNAs (snoRNAs) are a large class of short non-coding RNAs enriched in the nucleolus with a length of 60–300 nucleotides (nt), often regulating ribosomal RNAs (rRNAs) at the post-transcriptional level [4]. According to the specific nucleotide motif and the binding relationship with typical chaperone proteins, they are divided into box C/D snoRNAs (snoRD) and box H/ACA snoRNAs (snoRA) [5], guiding 2’-O-methylation and pseudouridylation of nucleosides, respectively [6, 7]. Remarkably, overwhelming evidence has shown that snoRNAs are involved in various tumors [8]. On the one hand, many dysregulated snoRNAs affect tumorigenesis and the development of cancer. For example, SNORA42 was significantly increased in oesophageal squamous cell carcinoma (ESCC) cell lines, as well as in tissues and serum of ESCC patients. Over-expression of SNORA42 significantly promoted the growth and metastasis of ESCC cells [9]; SNORD78 was upregulated compared with adjacent tissues in non-small cell lung cancer (NSCLC) patients. It could affect NSCLC cell cycle progression, and promote proliferation and invasion [10]; On the other hand, snoRNAs are stably present in various body fluids, including plasma, serum, and urine with the capability to distinguish between cancer patients and healthy individuals, revealing their potential as tumor biomarkers. For example, plasma snoRNAs (SNORD33, SNORD66, and SNORD76) were higher in NSCLC patients than those in the healthy controls and displayed promising diagnostic accuracy for NSCLC [11]. In addition, serum snoRNAs (SNORA2, SNORD12B, SNORA59B, SNORA70B, SNORD93, and SNORD116-2) were found to be closely related to the survival of ccRCC patients, empowering their potential as prognostic biomarkers [12].

Urine, produced in the kidney, is rich in proteins, DNAs, RNAs, specific antibodies, exfoliated cells, and other small molecules [13]. Owing to being readily available and truly non-invasive, substances in urine represented promising alternatives for disease diagnosis and monitoring [14]. In fact, non-coding RNAs in urine acted as tumor biomarkers have also been reported earlier. Urine miR-205 and miR-214 were significantly downregulated in prostate cancer (PC). Their sensitivity and specificity to distinguish PC from healthy controls were 89% and 80%, respectively [15]. However, there are few studies on urinary snoRNAs as biomarkers.

In the current study, we identified dysregulated snoRNAs in RCC tissues using TCGA and SNORic databases, confirmed the efficiency of SNORD15A, SNORD35B, and SNORD60 in urinary sediment (US) for RCC diagnosis as well as early diagnosis, revealing their roles in RCC tumorigenesis.

## Materials and methods

### Database

The expression profiles of snoRNAs in 516 RCC tissues and 71 adjacent tissues were downloaded from the online SNORic database (http://bioinfo.life.hust.edu.cn/SNORic). The clinical information of RCC patients included above was obtained from The Cancer Genome Atlas (TCGA) database.

### Patients and sample collection

Formalin-fixed and parrffin-embedded (FFPE) cancer tissues and paired adjacent tissues from 36 RCC patients, as well as the urine samples from 100 RCC patients and 131 healthy volunteers were collected from Shandong Cancer Hospital and Institute from April 2019 to January 2021. All patients didn’t receive any anti-tumor treatment before samples collection, or suffer any other endocrine, immune, or metabolic diseases. Clinical TNM stage was classified according to the American Joint Committee on Cancer (AJCC) eighth edition TNM stage, while grade was based on the 2016 edition World Health Organization (WHO) Furman grading system. The healthy donors showed no disease.

### RNA extraction and reverse transcription

RNAprep Pure FFPE kit (Tiangen Biotech, Beijing, China) was used to extract total RNA from FFPE tissue according to the protocol. Trizol reagent (Thermo Fisher Scientific, Waltham, MA, USA) was used to extract the total RNA of US. In brief, 15–20 mL of morning urine for each sample was collected from RCC patients followed by 3000 g for 20 min. Then the supernatant was removed and US at the bottom was washed twice with 1 x PBS. 1 ml of Trizol was added in each tube to extract total RNA according to the instructions. The reagent used to reverse transcribe was the Mix-X miRNA First-Strand Synthesis Kit (TaKaRa Bio, Nojihigashi, Kusatsu, Japan). The 10 µl reverse transcription system consisted of 5 µl buffer, 1.25 µl reverse transcription, and 3.75 µl RNA. The reverse transcription thermocycling program was as follows: 37 °C for 60 min followed by 85 °C for 5 min and 4 °C to the end. The final cDNA products were stored at -20 °C until use.

### Quantitative polymerase chain reaction (q-PCR)

LightCycler 480 qPCR system (Roche Diagnostics, Germany) was used for the q-PCR reaction. The reaction system contained 10 µl of SYBR Green Pro Taq HS Premix (AG11701, Accurate Biotechnology, Human China), 7.2 µl of RNase-free water, 0.4 µl of upstream and 0.4 µl of downstream primers, and 2 µl of cDNA template. The reaction program was set as follows: 95 °C for 30s, 95 °C for 5s, and 60 °C for 30s with 45 cycles. SnoRNA expression levels were normalized to the housekeeping gene U6 and measured by comparative cycle threshold (ΔCT). The formula was ΔCT = CT^snoRNA^-CT^U6^ as described previously [16]. Each sample was measured in duplicate. Primer sequences of the genes involved were listed in Table 1.

**Table 1:** Primer sequences of reference gene and snoRNAs.

Gene	Sequence (5’-3’)
U6-F	TGGAACGCTTCACGAATTTGCG
U6-R	GGAACGATACAGAGAAGATTAGC
SNORD15A-F	TTCGATGAAGAGATGATGACGAGTCTG
SNORD15A-R	CCACAGAACGCAGCACAGAGTAG
SNORD35B-F	TGGCAGATGATGTTTGTTTTCACGATG
SNORD35B-R	GCATCAGTTTTACCAAGTGGCTTTCTC
SNORD60-F	GTCTGTGATGAATTGCTTTGACTTCTG
SNORD60-R	GCCTCAGTCTTGCTAAATAATCAGACT

### Statistical analysis

Statistical analysis was performed by IBM SPSS Statistics 19 and GraphPad Prism 8.0.1 software. The Kolmogorov-Smirnov test was carried out to detect the distribution of data. For the comparison of the two groups, unpaired t-test was used if it met the normal distribution; if not, the Mann-Whitney test was used; For the comparison of paired samples, paired t-test or Wilcoxon rank test was used; For the comparison of multiple groups, the one-way ANOVA or Kruskal Wallis test was taken. Receiving operating characteristic (ROC) curve and the area under the curve (AUC) analysis were used to evaluate diagnostic efficiency of snoRNAs. Youden index (sensitivity + specificity − 1) was calculated to determine the best diagnostic efficiency with sensitivity and specificity [17]. All the results were represented as mean ± SD (Standard Deviation). Two-tailed p < 0.05 was defined as statistically significant.

## Results

### Identification of differential snoRNAs in TCGA database

**Fig. 1:**
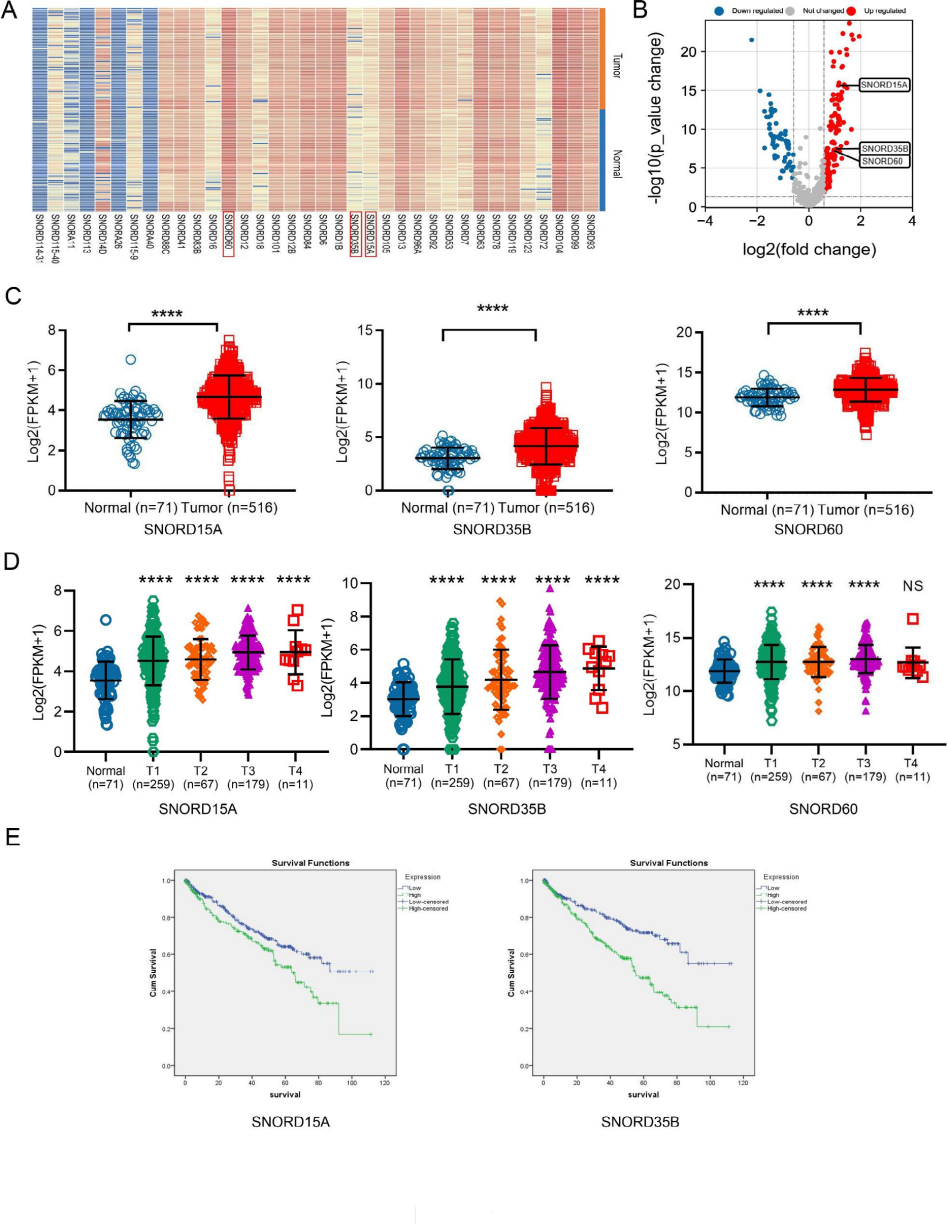
Identification of differential snoRNAs from datasets. **(A):** Heatmap showed up-regulated (marked in red) or down-regulated (marked in blue) snoRNAs in RCC tissues compared with adjacent normal tissues; **(B):** Volcano plots compared the expression fold-change of snoRNAs in RCC tissues vs. normal tissues; **(C):** Differential expression of SNORD15A, SNORD35B and SNORD60 in RCC tissues and adjacent normal tissues in TCGA; **(D):** Differential expression of SNORD15A, SNORD35B and SNORD60 between RCC tissues with different T stage and adjacent normal tissues in TCGA; **(E):** Kaplan-Meier survival curves of RCC patients with high- or low- expression of SNORD15A and SNORD35B, respectively; ****P < 0.0001; NS, no significance

To screen the differential snoRNAs, we downloaded the data of snoRNA expression profiles in RCC from SNORic database, including 516 cancer tissues and 71 control non-cancer tissues. SnoRNAs with differential expression between cancer and adjacent tissues were identified, and presented in heat-map and volcano map (Fig. 1A-B). We selected snoRNAs conformed to fold change > 1.5 and P < 0.05 for further study, and finally three snoRNAs: SNORD15A, SNORD35B and SNORD60 were determined as candidate genes.

As shown in Fig. 1C, SNORD15A, SNORD35B and SNORD60 increased significantly in cancer tissues (all, p < 0.0001) compared to those in normal tissues. Moreover, differential expression of these three snoRNAs was also observed among cancer tissues with different T stages (Fig. 1D). In addition, we analyzed the correlation between the expression of these three snoRNAs and the clinical characteristics of patients in TCGA. The expression of SNORD15A was related to the gender, age, T stage, TNM stage and grade of patients; SNORD35B was associated with age, T stage, distant metastasis, TNM stage and grade; SNORD60 was only related to grade but not to other factors (Table 2). The above results encourage us to explore the relationship between the three snoRNAs and the prognosis of RCC patients. Kaplan-Meier analysis showed that SNORD15A and SNORD35B other than SNORD60 were associated with the patient’s overall survival (OS) (Fig. 1E). Specifically, higher expression of SNORD15A or SNORD35B implied a shorter survival time, indicating their potential as prognostic indicators or therapeutic targets for RCC (p = 0.009, p < 0.0001, respectively).

**Table 2:** Correlation between snoRNA expression in TCGA and clinicopathologic characteristics of RCC patients

Characteristics		Cases	SNORD15A	SNORD35B	SNORD60
			**P Value**	**P Value**	**P Value**
Gender	Male	335	0.0109*	0.0701	0.0602
	Female	181			
Age	>=50	416	0.0443*	0.0253*	0.4483
	< 50	100			
T stage	T1	259	0.0023*	0.0001*	0.1796
	T2	67			
	T3	179			
	T4	11			
Lymph node metastasis	N0	228	0.9936	0.6330	0.9258
	N1	17			
	Not Available	271			
Distant metastasis	M0	406	0.3016	0.0062*	0.1947
	M1	78			
	Not Available	32			
TNM stage	I	253	0.0104*	0.0001*	0.1267
	II	55			
	III	123			
	IV	82			
	Not Available	3			
Grade	G1	13	0.0001*	0.0001*	0.0213*
	G2	218			
	G3	202			
	G4	75			
	Not Available	8			

* Bold value, p < 0.05

### snoRNAs: SNORD15A, SNORD35B and SNORD60 were upregulated in RCC FFPE tissues

**Fig. 2:**
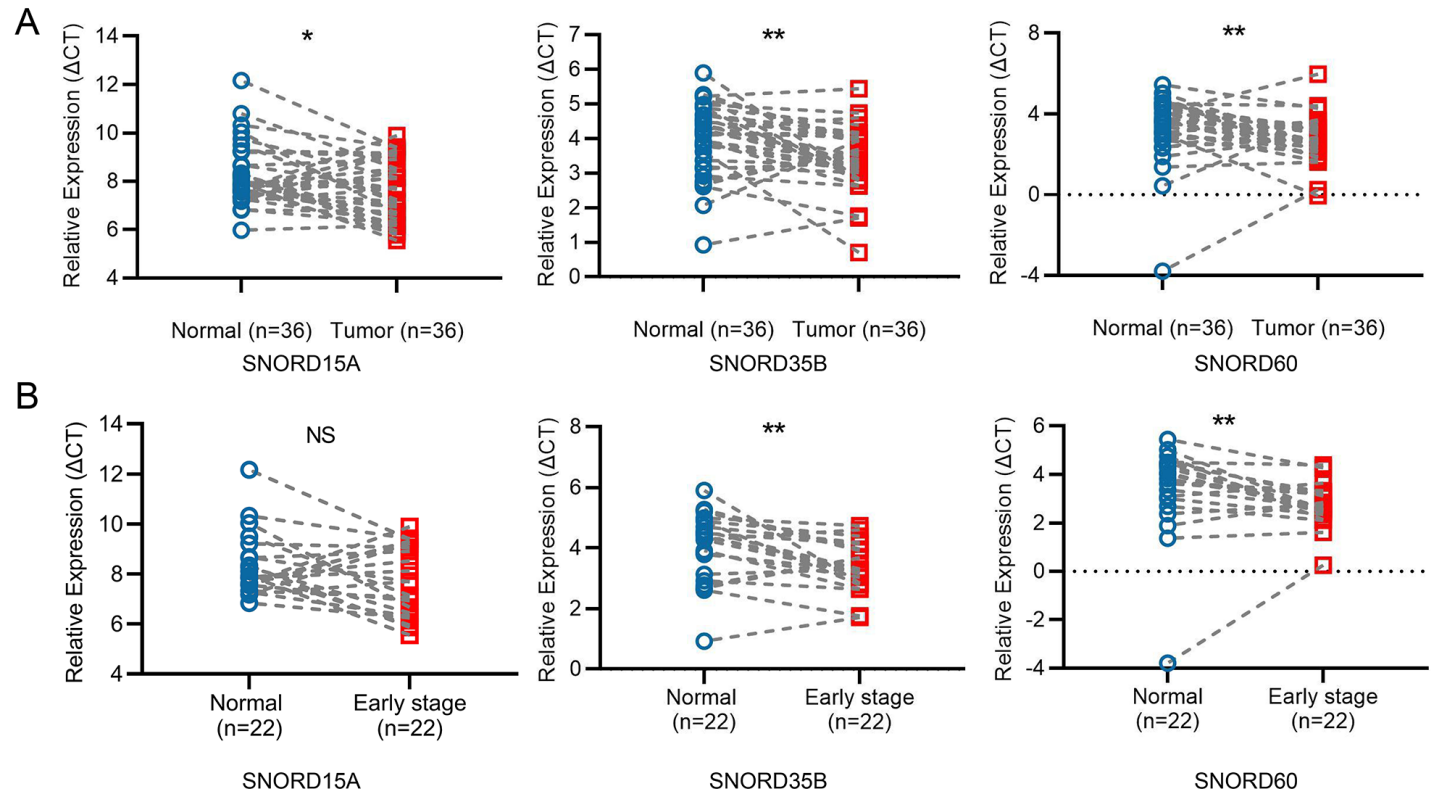
snoRNAs: SNORD15A, SNORD35B and SNORD60 were upregulated in RCC FFPE tissues. **(A):** SNORD15A, SNORD35B and SNORD60 were up-regulated in RCC FFPE tissues compared with para-carcinoma tissues (n = 36); **(B):** The expression of SNORD15A, SNORD35B and SNORD60 in early-stage RCC tissues and para-carcinoma tissues(n = 22); *P < 0.05; **P < 0.01; NS, no significance

To further verify the differences of SNORD15A, SNORD35B and SNORD60 between RCC and normal controls, 36 paired RCC FFPE tissues were collected and subjected to the q-PCR analysis. Consistent with the results from database, SNORD15A, SNORD35B and SNORD60 all appeared higher in cancer tissues (p = 0.0169, p = 0.0018 and p = 0.0049, respectively) when compared with the normal (Fig. 2A). Meanwhile, we also compared the expression level of these three snoRNAs in cancer and adjacent tissues from 22 pairs of RCC patients with early- stage (TNM stage I or II). As shown in the Fig. 2B, SNORD35B and SNORD60 also increased significantly in early cancer tissues (p = 0.0040, p = 0.0083, respectively), suggesting their potential roles in the early occurrence of RCC. However, SNORD15A did not show an expected result, which might attribute to the limited number of samples. The relationships between the three snoRNAs and clinical characteristics including gender, age, T stage, lymph node metastasis, distant metastasis, TNM stage and grade were also analyzed. As shown in Table 3, SNORD35B was related to TNM stage (p = 0.0086), but otherwise not related to others.

**Table 3:** Correlation between snoRNA expression in FFPE and clinicopathologic characteristics of RCC patients

Characteristics		Cases	SNORD15A	SNORD35B	SNORD60
			**P Value**	**P Value**	**P Value**
Gender	Male	20	0.6442	0.7683	0.7851
	Female	16			
Age	>=50	27	0.5284	0.5653	0.1932
	< 50	9			
T stage	T1	6	0.7391	0.2409	0.3939
	T2	10			
	T3	11			
	T4	9			
Lymph node metastasis	N0	21	0.7559	0.8559	0.8262
	N1	15			
Distant metastasis	M0	35	--	--	--
	M1	1			
TNM stage	I	13	0.4363	0.0086*	0.7427
	II	9			
	III	13			
	IV	1			
Grade	G1G2	24	0.5282	0.9200	0.4453
	G3G4	9			
	Not Available	3			

* Bold value, p < 0.05

### snoRNAs: SNORD15A, SNORD35B and SNORD60 as non-invasive diagnostic biomarkers for RCC

**Fig. 3:**
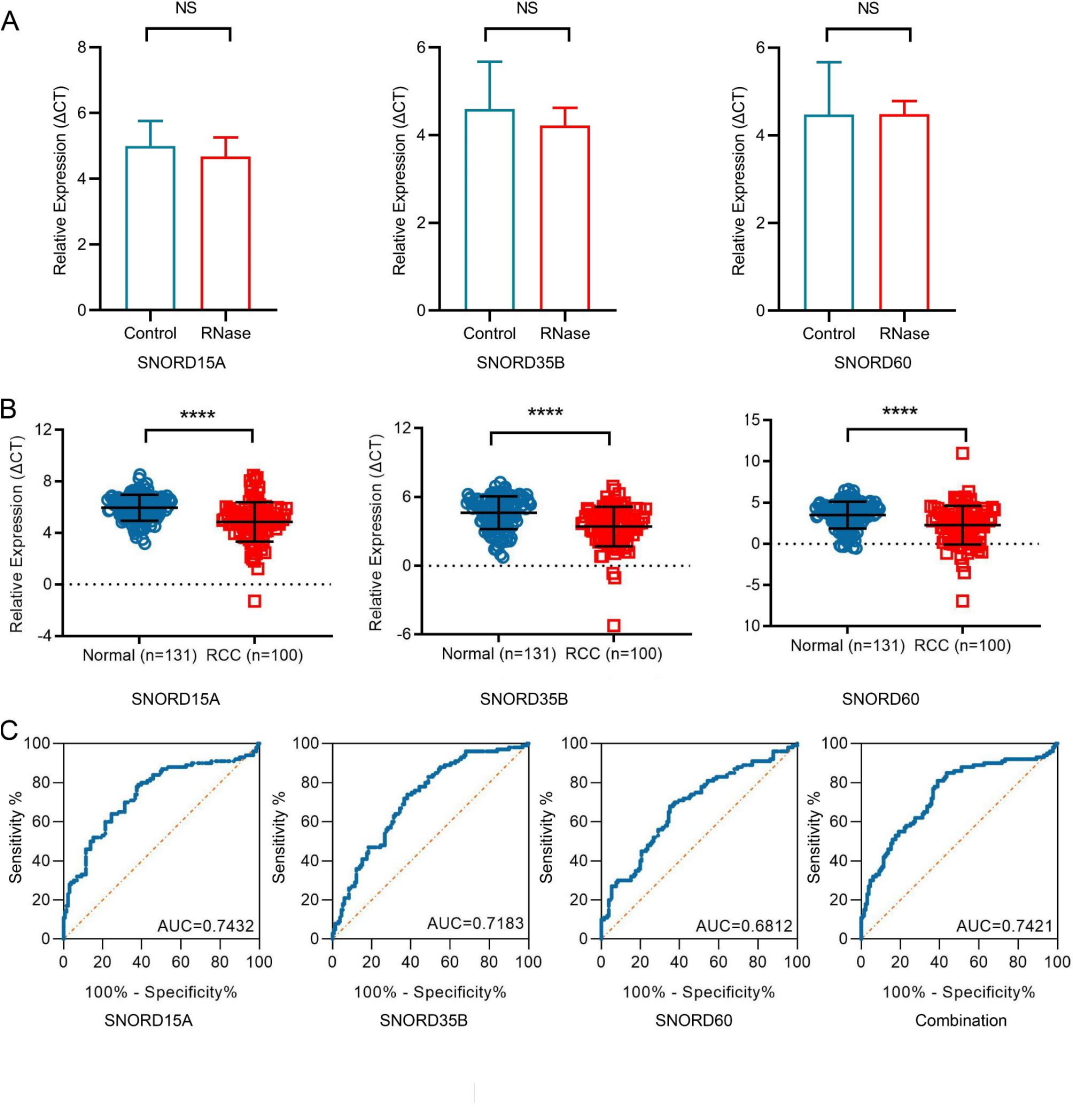
snoRNAs: SNORD15A, SNORD35B and SNORD60 as non-invasive diagnostic biomarkers for RCC. **(A):** Stability of snoRNAs in US: there was no statistical difference in the expression of SNORD15A, SNORD35B and SNORD60 between the control group (without RNase treatment) and the RNase group (with RNase treatment). **(B):** SNORD15A, SNORD35B and SNORD60 were up-regulated in US of RCC patients (n = 100) compared with healthy controls (n = 131); **(C):** ROC curve analysis of SNORD15A, SNORD35B and SNORD60 and their combination for RCC diagnosis. ****P < 0.0001; NS, no significance

We further studied whether SNORD15A, SNORD35B and SNORD60 in US could possess the potential as effective biomarkers for RCC diagnosis. First, we confirmed the stability of these snoRNAs in US. 14 US samples were randomly selected, and divided into two groups, treated with RNase A or not according to protocol. Notably, none of these three snoRNAs showed statistical differences between the two groups, indicating that snoRNAs exist stably in US (Fig. 3A).

Subsequently, urine samples from RCC cases (n = 100) and healthy individuals (n = 131) were introduced into the study. The expression of these three snoRNAs in two cohorts was detected and compared. As expected, the three snoRNAs were significantly up-regulated in US of RCC (Fig. 3B, all p < 0.0001). We also explored the relationship between expression of these three snoRNAs and clinical factors in 100 RCC patients. SNORD15A was correlated to TNM stage and smoking; SNORD60 was only associated with smoking but irrelevant to other characteristics, while the expression of SNORD35B was not associated with any clinical indicators (Table 4).

**Table 4:** Correlation between snoRNA expression in US and clinicopathologic characteristics of RCC patients

Characteristics		Cases	SNORD15A	SNORD35B	SNORD60
			**P Value**	**P Value**	**P Value**
Gender	Male	64	0.3018	0.3916	0.6384
	Female	36			
Age	>=50	78	0.9819	0.6020	0.7450
	< 50	22			
T stage	T1	55	0.2128	0.3201	0.8505
	T2	8			
	T3	29			
	T4	4			
	Not Available	4			
Lymph node metastasis	N0	87	0.9372	0.9275	0.7408
	N1	10			
	Not Available	3			
Distant metastasis	M0	75	0.6641	0.8185	0.5133
	M1	22			
	Not Available	3			
TNM stage	I	54	0.0322*	0.5360	0.6950
	II	3			
	III	18			
	IV	23			
	Not Available	2			
Grade	G1G2	49	0.2208	0.2173	0.4575
	G3G4	29			
	Not Available	22			
Pathological type	ccRcc	93	0.5670	0.9598	0.6738
	others	7			
Smoking	Yes	30	0.0436*	0.2735	0.0274*
	No	70			
Drinking	Yes	28	0.8115	0.8962	0.1924
	No	72			

* Bold value, p < 0.05; ccRcc, clear cell renal cell carcinoma

Then, ROC curve analysis was constructed to show their diagnostic performance. The AUC of SNORD15A was 0.7432 with 61.8% sensitivity and 79% specificity; AUC of SNORD35B was 0.7183 with 61.8% sensitivity and 74% specificity; AUC of SNORD60 was 0.6812 with 64.9% sensitivity and 68% specificity. When employed together, their combined diagnostic value showed that AUC was 0.7421 with 81% sensitivity and 61.1% specificity (Fig. 3C). These results indicate that these three snoRNA in US might be the novel biomarker for RCC diagnosis.

### snoRNAs: SNORD15A, SNORD35B and SNORD60 as early diagnostic biomarkers for RCC

**Fig. 4:**
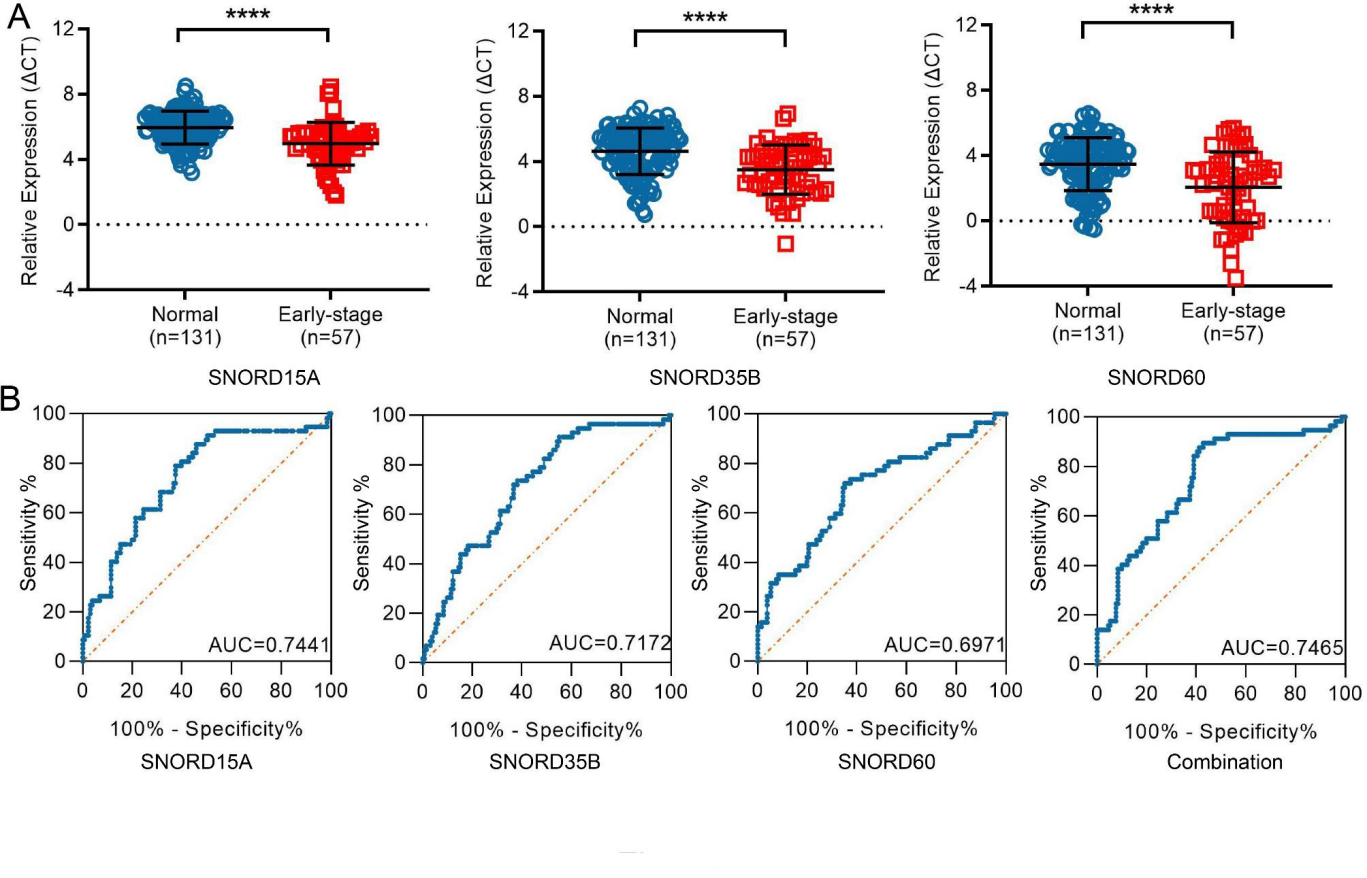
SNORD15A, SNORD35B and SNORD60 as biomarkers for early diagnosis of RCC. **(A):** SNORD15A, SNORD35B and SNORD60 were up-regulated in US of early-stage RCC patients (n = 57) compared with healthy controls (n = 131); **(B):** ROC curve analysis of SNORD15A, SNORD35B and SNORD60 and their combination for early-stage RCC diagnosis; ****P < 0.0001

As mentioned above, an effective early diagnostic biomarker was very crucial to improve the prognosis of RCC patients. Therefore, we next elucidated the efficiency of these three snoRNAs as early diagnostic biomarkers for RCC. As shown in Fig. 4A, the expression of SNORD15A, SNORD35B and SNORD60 in US of early-stage RCC patients was higher than that in healthy controls (all p < 0.0001). Their diagnostic performance was also evaluated by ROC. The AUCs were 0.7441 with 54.2% sensitivity and 87.7% specificity for SNORD15A, 0.7172 with 45% sensitivity and 91.2% specificity for SNORD35B, 0.6971 with 64.9% sensitivity and 71.9% specificity for SNORD60, respectively. When combined, they displayed a more optimal diagnostic value that AUC was 0.7465 with 89.5% sensitivity and 57.3% specificity (Fig. 4B). Taken together, these results suggest that SNORD15A, SNORD35B and SNORD60 possess potential to act as novel early diagnostic biomarkers for RCC.

## Discussion

RCC is currently the tenth most diagnosed tumour in women and the sixth in men. It makes up about 4% of all malignant tumours [18]. Despite the continuous progress of diagnosis and treatment technology, some patients were accompanied by tumor thrombus shedding and distant organ metastasis when diagnosed, and the prognosis was unfavorable [19]. Therefore, more sensitive and effective diagnostic biomarkers for RCC were urgently needed.

Recently, several studies have reported the effects of snoRNAs dysfunctions on colorectal cancer [20, 21], hepatocellular carcinoma [20], breast cancer [22] and prostate cancer [23], while our study clarified the potential functions of US snoRNAs: SNORD15A, SNORD35B and SNORD60 in RCC. SNORD15A, also known as U15A, is derived from the intron of Ribosomal Protein S3 (RPS3) with 148 nt and located on chromosome 11, targeting the A3764 site of 28 S rRNA; SNORD35B is produced from the intron of Ribosomal Protein S11 (RPS11) with 87 nt and located on chromosome 19, modifying the C4506 site of 28 S rRNA. Bioinformatics analysis showed that its target genes were significantly enriched in angiogenesis and Rap1 signaling pathway; SNORD60 is located on chromosome 16 with a length of 83 nt and is also targeted to modify 28 S rRNA. Actually, some researchers have studied their roles in diseases. Cornelia Braicu et al. reported that SNORD15A was an inhibitor during tumorigenesis of lung cancer [24]; SNORD35B was found to be up-regulated in SARS-CoV-2 infected A549 cells. It was considered to be involved in the immune response to virus invasion [25]. Different from the above research, our study revealed that these three snoRNAs were dysregulated in RCC. SNORD15A, SNORD35B and SNORD60 were significantly overexpressed in the cancer tissues as well as US of RCC, implying their potential role in RCC tumorigenesis.

More importantly, we demonstrated that snoRNAs in US other than in blood possessed promising potential as biomarkers for RCC diagnosis and early diagnosis. US might be the more favorable sample for the snoRNAs determination in RCC. It meets the fundamental conditions to act as the optimal biomarker. First, snoRNA expression in US was easily measured. It was convenient and efficient to detect the levels of three snoRNAs in US just using qPCR. Besides, snoRNA expression in US remained stable. No significant alternation was observed even treated by RNase A, which might attribute to the short length and the restriction sites protected by RNA binding protein (RBP) [26]. Second, aberrant expression of snoRNAs in RCC US empowered the potential to distinguish cancer and health. SNORD15A, SNORD35B and SNORD60 were significantly overexpressed in UC of RCC and early-stage RCC. Third, US possesses more considerable advantages than plasma or serum as tumor biomarkers. SnoRNAs in US had better primer specificity. We compared the amplification of the three snoRNAs in plasma, serum and US samples of RCC patients. The primer specificity of these three snoRNAs was better in US than that in the blood through qPCR and the agarose gel electrophoresis of qPCR products (data not shown), which might be due to the presence of abundant exfoliated tumor cells, resulting in the higher expression of the snoRNAs in US. For the reason the exfoliated RCC cells were enriched, US might be the better representation of RCC reality, it was able to collect a large number of changes of diseases and reflect minor pathological changes at the early stage. Forth, urine sampling was truly non-invasive. It could effectively avoid cross-infection and reduce the physical and psychological burden of patients, advocating the merit of urinary RNAs as more favorable diagnostic biomarkers for disease.

Several limitations should be carefully considered in the present study. Due to time constraints, the number of tissues and urine samples enrolled in this study were not large enough. In addition, we failed to obtain the test information of traditional tumor biomarkers such as carcinoembryonic antigen (CEA) in healthy controls, and the difference of diagnostic efficacy between these snoRNAs and the traditional biomarkers could not be judged. The prognostic information of these RCC patients was incomplete, and the potential of several snoRNAs as prognostic biomarkers cannot be evaluated accurately. These issues need to be further explored and addressed in the future research.

## Conclusion

In summary, our study revealed the important roles of SNORD15A, SNORD35B and SNORD60 in RCC. They were stable in US and could effectively distinguish RCC patients from healthy individuals. These three snoRNAs could be employed as novel biomarkers of early diagnosis and potential therapeutic targets for RCC.

## Data Availability

Please contact author for data requests.
